# Taking High-Tech to the Field: Leukemia Diagnosis in Pediatric Mexican Patients from Vulnerable and Remote Regions

**DOI:** 10.3390/diagnostics16030411

**Published:** 2026-01-28

**Authors:** Dalia Ramírez-Ramírez, Gabriela Zamora-Herrera, Rubí Romo-Rodríguez, Miguel Cuéllar Mendoza, Karen Ayala-Contreras, Enrique López Aguilar, Marta Zapata-Tarrés, Rosana Pelayo

**Affiliations:** 1 Laboratorio de Citómica del Cáncer Infantil, Centro de Investigación Biomédica de Oriente, Instituto Mexicano del Seguro Social, Puebla 74360, Mexico; josefina.ramirez@imss.gob.mx (D.R.-R.); gabriela.zamorah@alumno.buap.mx (G.Z.-H.); rubi.romo@imss.gob.mx (R.R.-R.); karen.ayalac@imss.gob.mx (K.A.-C.); 2 Comisión Coordinadora de Institutos Nacionales de Salud y Hospitales de Alta Especialidad (CCINSHAE), Secretaria de Salud, Mexico City 11400, Mexico; miguel.cuellar@salud.gob.mx; 3 Coordinación de Atención Oncológica, Instituto Mexicano del Seguro Social, Mexico City 07760, Mexico; javier.glopez@imss.gob.mx; 4 Secretaria de Salud, Mexico City 11400, Mexico; 5 Unidad de Educación e Investigación, Instituto Mexicano del Seguro Social, Mexico City 07760, Mexico

**Keywords:** acute leukemia, pediatric oncology, immunophenotyping, translational research, low- and middle-income countries

## Abstract

**Background/Objectives**: Acute leukemia, the most common childhood cancer, poses a significant public health challenge in low- and middle-income countries (LMICs) due to its high incidence and mortality rates. Survival rates in these regions are often lower, primarily due to delayed and inaccurate diagnoses, limited access to treatment, therapy abandonment, therapy-related toxicity, and inadequate healthcare infrastructure. In Mexico, a new initiative called OncoCREAN has been developed to address this urgent need by establishing local treatment centers near pediatric patients’ home cities, ensuring timely cancer detection and comprehensive disease treatment. **Methods**: A retrospective observational study was conducted on pediatric patients treated at the Mexican Social Security Institute (IMSS) between 18 May 2022 and 30 June 2025. Patients presenting clinical suspicion of acute leukemia were referred to OncoCREAN centers for sample collection and subsequent shipment to the Oncoimmunology and Cytomics Laboratory (OCL), where immunophenotyping confirmed the diagnoses. **Results**: The implementation of the OncoCREAN model significantly reduced diagnostic turnaround times, facilitating timely therapeutic decisions, minimized uncertainty, and optimized clinical management. The decentralized framework demonstrated feasibility across diverse geographic regions, ensuring access to advanced diagnostic technology for vulnerable populations and generating valuable data on disease incidence and molecular profiles. **Conclusions**: The OncoCREAN model highlights the critical importance of decentralizing high-technology diagnostic resources in modern pediatric oncology. This new approach to translational research that is accessible, inclusive, and relevant to society creates a paradigm shift in the management of childhood cancer and other diseases.

## 1. Introduction

### The Challenge of Leukemia Diagnosis in Resource-Limited Settings in Mexico

Acute leukemias (ALs) are the most common childhood cancer worldwide, accounting for 28.2% of cancer diagnoses in children and adolescents under 19 years old [1]. A significant disparity in leukemia incidence and survival rates among children and adolescents is observed between high-income countries (HICs) and LMIC, economies with a gross national income (GNI) per capita lower than USD 13,935 [2]. In countries like Canada, Cyprus, Belgium, and Denmark, survival rates reach up to 85%, whereas in LMICs, they drop to as low as 46% [3]. In developed countries, the childhood leukemia incidence has increased, while mortality rates have declined due to advancements in diagnostic and treatment capabilities [4].

Each year, it is estimated that approximately 430,000 individuals under 20 years of age are diagnosed with cancer, with only 10.5% of cases occurring in HICs, compared to 89.5% in LMICs [5]. Mexico is a country with substantial economic disparities; however, it is classified as a middle-income country (MIC) by the World Bank [2]. Mexico has a population of 130,861,007 inhabitants (2024) [6], with an incidence of cancer estimated at 16 cases per 100,000 in individuals between 0 and 19 years, with leukemia being the most frequent malignancy. Leukemia incidence and mortality rates are 5.5 and 2.5 cases per 100,000, respectively, exceeding global averages by approximately 1.8-fold and 2-fold [7].

In Mexico, more than 50% of childhood cancer cases are diagnosed as leukemia [1]. Mexican pediatric leukemia patients face considerable challenges, including a higher prevalence of biological and environmental factors associated with a poor response to treatment, increased relapses, treatment-related toxicity, and treatment abandonment rates that can reach as high as 50% in some remote and vulnerable regions of the country [8].

Several health institutions in Mexico lack state-of-the-art diagnostic services or offer limited clinical services, access to specialized pediatric oncologists, and critical care training for providers. The early and accurate diagnosis of AL is key for risk stratification, appropriate therapy selection, and the timely initiation of treatment, improving survival rates [9]. Strikingly, the geographic, economic, and cultural diversity of Mexico impacts healthcare, particularly in terms of access to diagnostic and treatment services. Therefore, providing a universal and unified service with the best quality standards is a priority.

We have installed an OCL to exclusively provide precise diagnoses and treatment response monitoring through immunophenotyping by flow cytometry for pediatric patients from remote and vulnerable regions, especially in the states of Puebla, Tlaxcala, and Oaxaca, where a significant proportion of the population lives in poverty, and a very high incidence of AL in children is reported [9]. To expand access to specialized medical services nationwide, the Mexican Social Security Institute (IMSS), the main social security institution covering over 75 million individuals, launched the OncoCREAN strategy (Statal Reference Centers of Pediatric Oncology). This model originated from the urgent need for onco-pathology services in states with limited access to healthcare services [10,11]. Previously, families had to travel to large cities like Mexico City for diagnosis and treatment, implying additional expenses and leading to significant delays that contributed to high rates of treatment abandonment and relapse. Today, OncoCREAN has established 35 centers, with at least one in each state of the country, strategically located to provide specialized medical services to children and adolescents with cancer closer to their homes. These centers aim to reduce diagnostic delays and improve access to timely treatment, ultimately increasing the life expectancy of pediatric patients. The implementation of this strategy was possible due to the extensive IMSS infrastructure across the country [10,12].

Here we present a narrative description of the steps involved in creating OncoCREAN, including the processing and shipment of samples, the training of personnel responsible for caring for patients with AL, and ensuring follow-up according to the Total Therapy XV protocol, as well as the early management of complications and the provision of adequate physical spaces in hospitals. Finally, as part of these efforts, we present preliminary results from the epidemiological analysis of patients at debut and follow-up at the OCL between 2022 and 2025.

## 2. Materials and Methods

A retrospective observational study was conducted on pediatric patients treated at the IMSS from 18 May 2022 to 30 June 2025, who presented clinical suspicion of acute leukemia and confirmed by immunophenotyping at the OCL. Patients with suspected leukemia were referred to OncoCREAN centers for sample collection and subsequent shipment to the OCL. Following the issuance of the corresponding diagnostic report to the treating physicians, patients received a compatible diagnosis and initiated treatment according to the assigned risk stratification, as outlined by López-Aguilar et al. [13]. This study included cases from 29 of the 32 Mexican states; Aguascalientes, Baja California, Baja California Sur, Campeche, Ciudad de México, Chiapas, Chihuahua, Coahuila, Colima, Durango, Estado de México, Guanajuato, Guerrero, Hidalgo, Michoacán, Morelos, Nuevo León, Oaxaca, Puebla, Querétaro, Quintana Roo, San Luis Potosí, Sinaloa, Sonora, Tabasco, Tamaulipas, Veracruz, Yucatán, and Zacatecas.

## 3. Results

### 3.1. Key Strategic Actions to Improve Leukemia Diagnosis

Taking high-tech diagnosis to the field for pediatric patients with leukemia in Mexico involves several considerations and challenges that can significantly improve prompt and accurate diagnoses, benefiting treatment outcomes.

**Figure 1:**
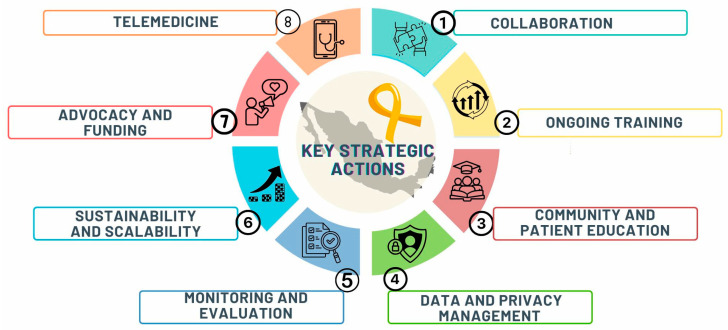
**Key strategic actions to improve leukemia diagnosis within OncoCREAN**. To improve access to pediatric cancer diagnosis and care in Mexico, punctual strategies have been developed, including the following: (1) collaboration, (2) ongoing training, (3) community and patient education, (4) data and privacy management, (5) monitoring and evaluation, (6) sustainability and scalability, (7) advocacy and funding, and (8) telemedicine.

The OncoCREAN strategy includes eight key actions (Figure 1).

#### 3.1.1. Collaboration with Local Healthcare Providers Through the Establishment of a Network with Hospitals, Clinics, and Healthcare Professionals to Ensure Effective Referral and Follow-Up Care Based on Field Diagnoses

The initial contact for suspected AL cases occurs at OncoCREAN centers in every state, where hematologists and/or oncologists identify potential cases, collect bone marrow (BM) samples or, in exceptional cases, peripheral blood, and request immunophenotyping tests to confirm the disease. An electronic request is submitted to OCL personnel, including details of the responsible physician, patient information, clinical data, sample information (number of tubes, aspiration date) and the type of test, diagnosis, or measurable residual disease (MRD). Once the sample is collected, it is dispatched within 24 h. A standardized protocol for correct sample packing is shared with the center, ensuring the samples are placed in triple-layer packaging at ambient temperature. Both diagnostic and treatment monitoring samples are sent to the OCL for processing. Currently, 32 centers across 29 states submit samples for evaluation. The OncoCREAN reference center, through the OCL, has developed *The Guide for Sample Shipment: Immunophenotype and Minimal/Measurable Residual Disease Study* and disseminated to its 32 affiliated centers (Supplementary Materials). Likewise, the requirements for sample collection in patients with suspected childhood leukemia are aligned with the institutional algorithms and clinical practice guidelines established by the Mexican Social Security Institute and the Ministry of Health.

#### 3.1.2. Ongoing Training of Healthcare Providers to Ensure That They Fully Understand and Effectively Use These New Technologies, Including Operations, Interpretation of Results, and Data Management to Achieve Process Automation

To take advantage of new technologies in the diagnosis, prognosis, and monitoring of childhood disease, it is essential that all professionals directly or indirectly involved in patient care are highly competent. This includes the medical personnel responsible for ordering the appropriate tests at the right time and interpreting the results, which is critical for accurately stratifying patient risk both at diagnosis and during treatment monitoring. In cases where re-stratification is required, these professionals play a key role in adjusting treatment dosages, reducing them for low-risk patients and increasing them for those at higher risk. Equally important are the professionals responsible for operating high-tech equipment, analyzing data, and interpreting results. A crucial factor in the successful implementation of this strategy was the alliance with the OCL, which currently serves as a reference laboratory for cytometric diagnosis, covering most of the country. Highly trained professionals supervise sample processing and interpret results, strictly adhering to EuroFlow™ standardized protocols for both instrumentation and sample handling.

One of its key achievements is delivering patients’ reports to attending medical professionals within 72 h of sample arrival, significantly reducing diagnosis times and facilitating timely therapeutic decisions [9]. As a result, this strategy has standardized diagnostic, prognostic, and treatment response monitoring for pediatric leukemia nationwide. Ongoing courses and workshops are critical to ensure the continuous updating of knowledge.

#### 3.1.3. Community and Patient Education: Educate Communities and Patients on the Benefits of Early Diagnosis and the Use of High-Tech Tools, While Addressing Any Cultural or Language Barriers That Could Affect Understanding and Acceptance

In rural areas, geographic and economic characteristics often present unique challenges that can conflict with the introduction of new technologies. While these initiatives aim to provide theoretical benefits to the community, they often generate resistance to change. It is crucial to recognize that educating the community and patients plays a fundamental role in understanding and accepting these new technologies and approaches. This educational role helps transform the health sector and can influence local cultures. Creating an innovative mindset represents a paradigm shift within a community, and as this shift extends to other areas, it can transform entire regions, facilitating the adaptation and acceptance of new approaches.

#### 3.1.4. Data and Privacy Management by Implementing Protocols for Secure Collection, Storage, and Transmission of Data to Protect Patient Privacy and Ensure Compliance with Local Regulations

The use of a research center for healthcare purposes has represented a paradigm shift, which gained prominence globally during the COVID-19 pandemic. In the case of cancer care, particularly childhood cancer, several countries have established institutions to certify and validate the diagnosis of some pathologies. Examples include the Children’s Oncology Group (COG) in the United States or the Programa Infantil Nacional de Drogas Antineoplásicas (PINDA) in Chile. In Mexico, however, there had been no prior collaborative effort of this kind until the launch of this initiative, which aims to improve outcomes through the homogenization of diagnostic techniques—an essential first step. Achieving compliance with regulations related to data management, information collection, safeguarding, and data protection presents significant challenges, especially when involving multiple hospitals and even more so on a national scale. The initiative of diagnosing childhood leukemia in a research center has been recognized by the IMSS authorities as a permanent benefit for children. This approach serves as a model for managing similar strategies for other diseases.

#### 3.1.5. Monitoring and Evaluation: Establish a System to Monitor the Impact of Field Diagnostics on Patient Outcomes

The goal of this leukemia diagnostic center is to implement personalized treatment strategies from the first point of patient contact and monitor patients through MRD tests. This approach aims to enable personalized treatment, identifying cases suitable for immunotherapy, those requiring hematopoietic stem cell transplantation, and patients who do not respond to treatment and may be candidates for palliative care. This would allow for the tracking of childhood leukemia incidence, treatment initiation times, and survival. With this data, it will be possible to improve patient outcomes through the implementation of public health policies.

#### 3.1.6. Sustainability and Scalability to Ensure That the Initiative Is Sustainable Long-Term and Can Be Scaled up to Reach More Regions and Patients Across Mexico

In Mexico, this marks one of the first efforts to establish a national network for diagnostics, monitoring, and follow-up. Over the past two years, resources from the IMSS and the National Council of Humanities, Sciences, and Technologies (CONAHCYT) have supported the creation of these centers and labs, building experience in managing samples, equipment, and personnel. The next steps involve scaling this approach to other neoplasms and regions across Mexico, which will help to provide better attention for the Mexican population and to identify and resolve region-specific challenges.

#### 3.1.7. Advocacy and Funding: Secure Government Support and Funding, Along with Partnerships with NGOs and Private Sector Entities, to Sustain and Expand the Program

The establishment of comprehensive governmental and private sector funding mechanisms, complemented by strategic NGO partnerships, to guarantee the long-term sustainability of the program necessitates the development of a resilient financial ecosystem that integrates institutional capacity, private sector technological expertise, and NGO outreach. Efforts must specifically target the resources required for the immediate procurement of high-tech diagnostic equipment, specialized technical training for field deployment, and the logistical scaling of these advanced tools to reach pediatric leukemia patients across remote areas.

#### 3.1.8. Integration with Telemedicine for Remote Consultation with Laboratory Specialists Who Can Provide Guidance Based on the Field Diagnosis

The AL diagnosis program started as a research initiative in central Mexico targeting the most vulnerable States, and, over time, it gradually expanded to the rest of the country. Telemedicine has played a crucial role in facilitating communication between specialist physicians, couriers, laboratories, and all personnel involved in the process. It has also facilitated the discussion and resolution of complex cases and controversial results.

### 3.2. Taking High-Technology Leukemia Diagnosis and Prognosis to Vulnerable and Remote Regions

To address the challenge of bringing high-tech diagnosis to the field for pediatric patients, recognizing AL as a complex disease, an extensive study is required. Thus, the OCL was established through the joint effort of IMSS and the CONAHCYT as part of the National Project for Research and Incidence (PRONAII) of Childhood Leukemias, with the mission of providing precision diagnosis and treatment monitoring through immunophenotyping in vulnerable regions of the country [14]. This high-tech laboratory, equipped with flow and mass cytometry, was established in a rural region to initially benefit marginalized populations from Puebla, Tlaxcala, and Oaxaca [14,15]. As stated before, only three years of operation have been enough to achieve significant results, including the delivery of detailed patient reports to the responsible medical professional within 72 h upon sample arrival and the identification of a distinct profile associated with an increased risk of persistent disease after treatment [16]. This risk stratification was achieved through a comprehensive analysis of the tumor, immune system, and microenvironment components, aiming to understand the complex biology of acute leukemia [15,16].

Strikingly, each step of the process is meticulously managed once the patient’s samples arrive: the accurate completion of requisition forms by the medical doctor, quality control of the samples, standardization of flow cytometry equipment and sample processing, classification of AL cases, and further reporting to the responsible health center.

During the analytical phase, and as a part of the EuroFlow protocols, rigorous quality controls have been implemented through the full standardization of the flow cytometry methodology for the diagnosis and monitoring of hematologic diseases. These include the following:

- Standardized Operating Procedures (SOPs): The consortium developed uniform and detailed technical protocols for sample preparation, instrument setup and calibration (flow cytometers), and data analysis [17].
- Harmonized Antibody Panels: EuroFlow™ has defined scientifically validated and standardized antibody panels that are essential for ensuring the reproducibility of results across different laboratories [18].
- Multicenter Evaluation: The reproducibility of results obtained with these panels and protocols is ensured through multicenter evaluations, in which multiple laboratories participate to ensure methodological consistency.
- Computational Tools and Automation: The use of advanced computational tools and automated data analysis has been fundamental for efficiently managing large data volumes and ensuring reproducible results [19].

**Figure 2:**
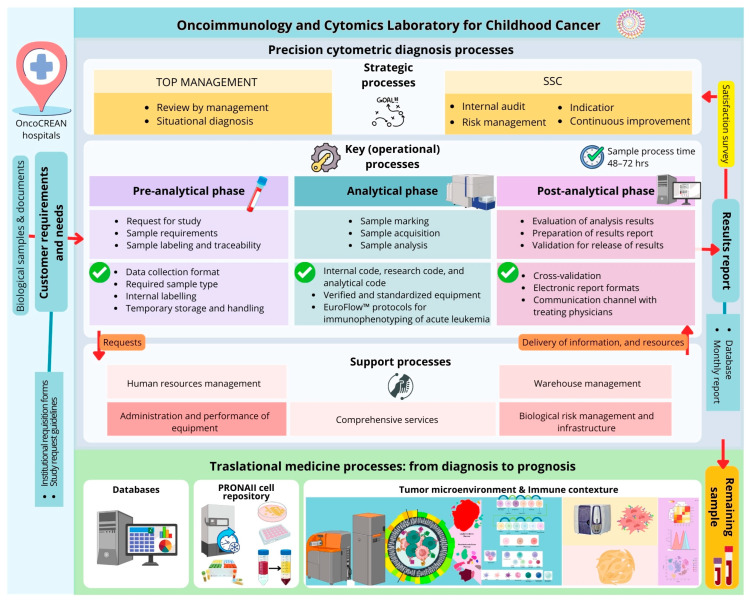
**From diagnosis to prognosis processes.** When clinicians at OncoCREAN hospitals suspect a case of leukemia or need to monitor a patient’s treatment response, a two-way communication process begins. After filling out requisition forms and following request guidelines, biological samples are transported to the Oncoimmunology and Cytomics Laboratory for Childhood Cancer (OCL) for precision cytometric diagnosis processes, which are divided into three categories: (1) strategic processes, involving top management and internal evaluations for quality assurance and continuous improvement; (2) key (operational) processes, where the sample undergoes pre-analytical, analytical, and post-analytical phases within 72 h, after which a result report is sent to the responsible medical professionals; and (3) support processes, which include all administrative and additional activities to ensure the proper functioning of the operational processes. Remaining samples are then incorporated into translational medicine processes, where different components are collected and preserved as part of the PRONAII cell repository; these components are also used to assess the tumor microenvironment and immune contexture of the patients through high-tech platforms like mass cytometry or the formation of ex vivo organoids, to provide prognosis information. Data from diagnosis to prognosis processes is collected in databases that are later used to generate complex analysis and disease models.

A remarkable characteristic of the OCL is the focus on translational medicine. With the remaining samples, a specimen repository is created, which includes both soluble and cellular components (plasma, hematopoietic cells, and mesenchymal stromal cells) and facilitates in-depth research of the tumor biology, immunology, and microenvironmental contexture of the disease [9]. Databases are continually updated with clinical, socioeconomic, macro- and microenvironmental, tumoral, and immunological immunophenotype information to create an Atlas of Mexican childhood leukemia (Figure 2).

**Figure 3:**
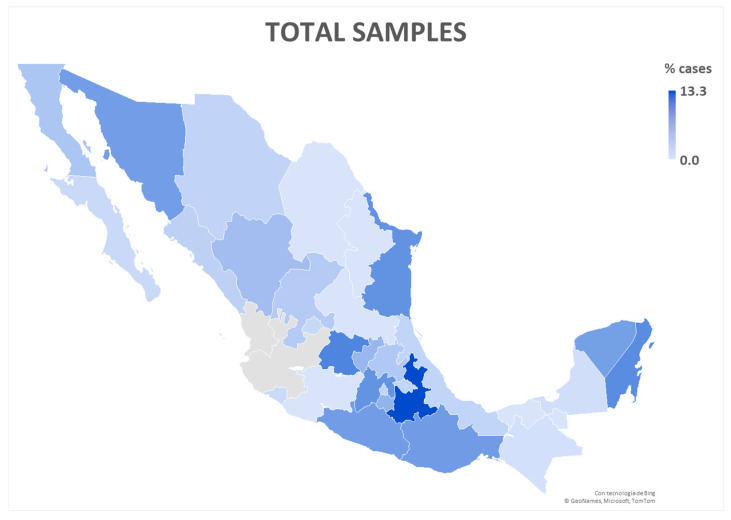
**Geographic distribution of pediatric leukemia diagnoses in Mexico.** Percentage of confirmed hematological malignancy diagnoses (B-ALL, T-ALL, AML, ALAL/MPAL, and MDS) in pediatric patients (0–18 years) per Mexican state. Data reflects cases confirmed by immunophenotyping and referred to the Oncoimmunology and Cytomics Laboratory. The blue gradient indicates the proportion of the total national cases received by each state’s laboratory, where the darkest shade represents the largest number of samples compatible with acute leukemia and/or follow-up by MRD studies. States shown in grey do not send samples to OCL.

**Figure 4:**
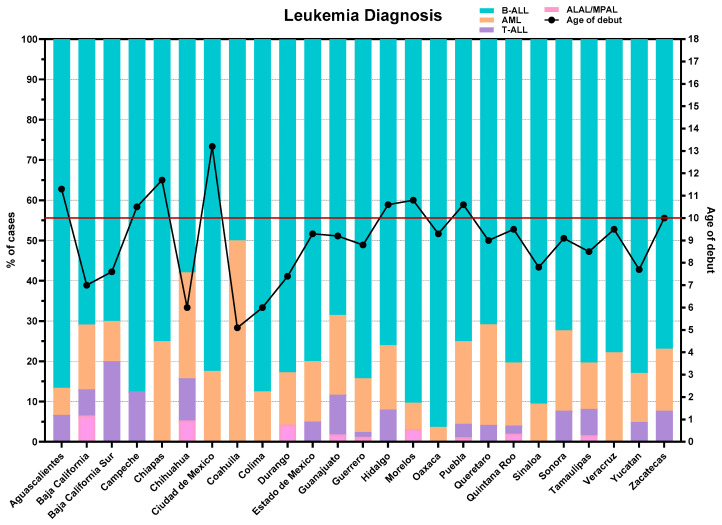
**Distribution of acute leukemia cases by state.** The bar chart depicts each federal entity along with the relative proportions of acute leukemia subtypes (B-ALL, T-ALL, AML, and ALAL/MPAL). The secondary vertical axis (right) displays the mean age at diagnosis, providing an integrated view of both the epidemiological distribution and age-related trends across regions. The red line indicates the age threshold of 10 years, above which B-ALL diagnoses are associated with a poorer prognosis due to a higher probability of measurable residual disease (MRD) detection.

Under this new model of inclusive precision care, we processed 3540 samples between May 2022 and June 2025 (Figure 3). Although the standardized immunophenotype diagnosis has revealed that B-cell precursor acute lymphoblastic leukemia (B-ALL) is the most frequently identified subtype, increasing numbers of other leukemia subtypes in adolescents are recorded in vulnerable regions (Figure 4). Notably, the states of Guanajuato, Puebla, and Guerrero have sent the highest number of samples for AL confirmation. Among the received samples, only 45 (1.3%) were non-evaluable for result interpretation, in which case the pediatric hematologist or oncologist was notified, and a new BM aspiration was requested for processing.

The regional distribution of lymphoid leukemia subtypes across Mexico reveals striking epidemiological patterns. In the northwestern states (Baja California, Baja California Sur, Chihuahua, Durango, Sinaloa, and Sonora), there is a discernible increase in T-lineage (7.4%) and mixed-phenotype leukemias (2.7%), occurring at the expense of B-ALL (74%) (Figure 4). In contrast, Acute Myeloid Leukemias (AMLs) exhibit a marked concentration in eastern Mexico, particularly in Puebla, Veracruz, and Hidalgo, with a mean prevalence of 19.6% (Figure 4). This elevated proportion is of particular concern for the health sector, as AML—although less frequent than B-ALL, which represents approximately 80% of the newly diagnosed AL in Mexico—shows an atypical epidemiological profile compared to global statistics, with a disproportionately higher incidence among adolescents (over 10 years old).

In the case of B-ALL, a polarization of incidence has been observed in the southwest of the country (Guerrero, Oaxaca, and Chiapas) (Figure 4), federative entities that are severely impacted by a low economic income [20] as well as complications in accessing the health system.

Due to these challenges, the OncoCREAN centers were established to improve pediatric cancer care. These centers offer an integral model that ensures rapid diagnosis and specialized treatment, encompassing everything from timely detection to high-complexity care, along with psychological and social support for patients and families, ultimately aiming to increase survival rates. As a direct outcome of these efforts and the adoption aiming of advanced diagnostic strategies, we have reduced the turnaround time for delivering results to physicians by over 70%, thereby facilitating timely therapeutic decisions, reducing uncertainty, and enhancing the overall efficiency of clinical management. This valuable data offers critical insights for evaluating and implementing better public health surveillance strategies. For instance, early mortality was significantly lower among patients who underwent MRD monitoring at the OCL compared to those who did not. Furthermore, overall, the one-year survival rate was also higher in patients evaluated at the OCL (89.6% vs. 75.2%, *p* < 0.001) [21].

**Figure 5:**
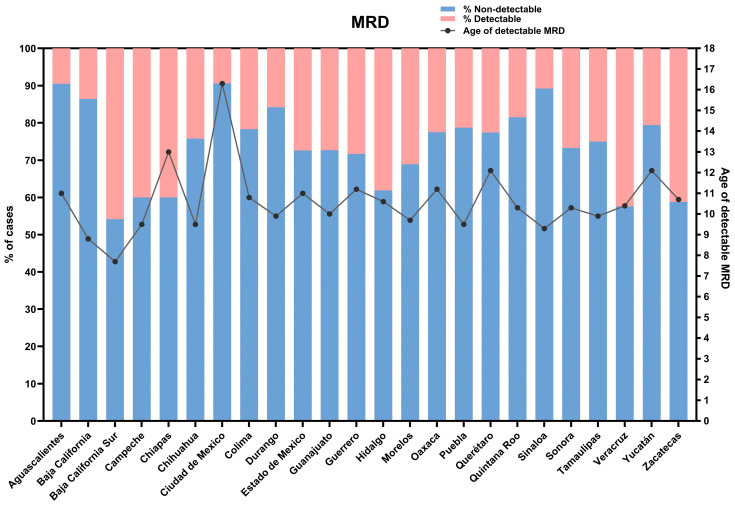
**Representation of measurable residual disease (MRD) across Mexican states.** The bar chart illustrates the distribution of MRD-positive cases, while the secondary vertical axis (right) indicates the mean age at which MRD was detected. This dual representation integrates geographic variation with age-related patterns of MRD presentation.

In addition to regionalization, the national data compiled thus far has enabled us to identify the federative entities with a higher potential risk for early relapses (Figure 5). While clinical risk stratification is conventionally based on age, our immunotumoral and microenvironmental findings in B-ALL have allowed us to uncover potential underlying mechanisms in a specific group of children older than 10 years. These patients are associated with a six-fold increase in the risk of developing MRD [16]. In Mexico, eight states show a higher incidence of B-ALL, where the median age at diagnosis surpasses 10 years (Figure 4). This observed age profile strongly suggests a greater potential for early relapse. Consequently, this analysis is crucial for supporting more precise clinical risk stratification and advocating for intensified surveillance protocols to significantly reduce the probability of detectable MRD [16].

Beyond the monitoring of initial diagnoses through the OncoCREAN strategy, longitudinal patient follow-up via MRD assessments (Figure 5) has enabled the identification of specific entities and geographic regions that demand intensified clinical attention to reduce the prevalence of detectable MRD. Notably, the states of Hidalgo, Veracruz, and Zacatecas exhibit an average detectability rate of 35.1%, underscoring the urgent need for targeted interventions. The integration and cohesion of more comprehensive approaches, such as those outlined in this manuscript, constitute essential responsibilities for decision-makers and public health policy promoters. Ensuring rigorous adherence to referral pathways, diagnostic accuracy, therapeutic protocols, follow-up, and surveillance in pediatric patients is imperative to mitigate preventable mortality and to strengthen equity in childhood cancer care across diverse healthcare settings.

## 4. Discussion

### The Future of Pediatric Leukemia Diagnosis in Mexico: A Roadmap to Widespread Implementation

Latin America and the Caribbean are among the regions with the most inequity in childhood cancer survival [22]. The Global Initiative for Childhood Cancer in Latin America (GICC) provides a global and regional framework for improving childhood cancer, and OncoCREAN and its eight actions (Figure 1) are aligned with the implementation of this approach in Mexico: a national network that operationalizes the CureAll pillars through standardization, capacity building, surveillance, and inter-institutional coordination [23].

The incidence and mortality of childhood leukemia vary widely by region; approximately 84.1% of childhood cancer cases are anticipated to occur in LMICs, driven by factors such as population growth, socioeconomic disparities, environmental changes, and lifestyle factors [4]. A phenomenon associated with access to health services is that while cancer incidence rates are higher in urban areas, mortality rates are higher in rural areas; reported factors that contribute to this are that patients in rural areas are frequently diagnosed at later stages; are less likely to receive standard treatments, optimal medical follow-up, or support services; and consequently experience worse health outcomes than urban patients [15,24]. Treatment abandonment is a common cause of treatment failure in LMICs; the main reasons for this are families with lower incomes and poorer educational backgrounds [25,26]. The reasons for treatment abandonment include poverty, a lack of interest in their own disease, cultural myths, feelings of guilt, and social discrimination among their peers. These disparities reflect broader social and structural barriers that shape how families navigate the diagnostic and treatment process. In this context, caregivers facing socioeconomic hardship or limited health literacy often experience additional challenges in understanding medical information, engaging with care teams, and sustaining long-term treatment adherence [27]. A prospective cohort study of 574 patients under 18 years diagnosed with AL, conducted in Oaxaca, Puebla, and Tlaxcala from 2021 to 2023, showed that living ≥141 km from a hospital (adjusted OR = 1.68; 95% CI: 1.02–2.74; *p* = 0.03) was significantly associated with an abandonment of treatment [28]. A retrospective analysis of Mexico’s Seguro Popular program (2005–2015) reported a 5-year overall survival of 61.8%, with wide state variations (74.7–43.7%). The mortality risk increased in non-pediatric hospitals (HR = 1.18; 95% CI 1.09–1.26), centers without pediatric oncology/hematology specialists (HR = 2.17; 95% CI 1.62–2.90), and low-volume hospitals (HR = 1.22; 95% CI 1.13–1.32). While Sinaloa, Michoacán, and Zacatecas exceeded 70% risk-standardized survival, seven states reported a survival of 50% or lower (Hidalgo, Puebla, Chiapas, Tabasco, Oaxaca, Veracruz, and Campeche) [29], which is consistent with our results showing that Hidalgo, Chiapas, Veracruz, and Campeche are the states with the highest percentage of detectable MRD (Figure 5).

A high proportion of the population in the states of Puebla and Oaxaca lives in poverty (62.4% and 61.7%, respectively) at frequencies that are higher than the national average (36.3%). The Mixteca region, an area connecting the capital cities of these states, showed an AL incidence rate of 96.7 and 88.5 cases per million, for groups 0–14 and 0.19, respectively [15]. Another study showed consistent findings regarding socioeconomic conditions in Mexico City and AL. They observed the highest rates of AL in municipalities located mainly in the east of Mexico City, which are characterized by a low socioeconomic status (SES). On the contrary, low incidence rates of AL were observed in municipalities with a better SES. SES cannot be separated from the ethnic composition; the lower the SES of a Mexican individual, the higher the probability of having an Amerindian ancestry [30]. Moreover, children residing in lower SES neighborhoods are significantly more likely to require ICU admission at the time of leukemia diagnosis compared with those living in higher SES areas, underscoring the clinical impact of socioeconomic disparities [31]. Additionally, the nutritional status at the time of diagnosis has emerged as a lifestyle factor associated with leukemia outcomes. Epidemiological evidence suggests that obesity may have an association with worse outcomes and increased mortality in leukemic patients. The main pathophysiological pathways include bone marrow adipose tissue, pro-inflammatory cytokines, oxidative stress, and others [32]. In Mexico, over one-third of children aged 5–11 years are overweight or obese, posing a significant public health concern and a potential risk factor for adverse leukemia outcomes [33]. By contrast, Guzmán León et al., in a retrospective-observational study in Mexico, showed that patients with ALL and malnutrition had a significantly lower survival rate according to their BMI (76% vs. 63%, *p*  =  0.049) [34].

Research on environmental factors associated with pediatric leukemia has often examined exposures in isolation, limiting our understanding of what is clearly a multifactorial disease process. Some studies have reported a higher childhood ALL incidence in urban compared with certain rural areas of the United States [35]. Similarly, studies by González-García et al. identified sex- and region-specific patterns, including a higher proportion of girls affected at ages 0–4 and survival differences among boys aged 0–14 between rural and urban settings, highlighting areas that require further investigation [36].

These findings can be interpreted in light of the model proposed by Greaves, who suggests that childhood ALL could be considered a paradoxical consequence of progress in modern societies, where lifestyle changes have limited the early exposure to microorganisms. This restriction creates complications in the evolutionary adaptation of the immune system and contemporary lifestyles, contributing to the risk of leukemogenesis [37], reinforcing the need to consider environmental exposures as interconnected components within broader biological and social systems.

Maternal prenatal exposures have gained relevance following evidence of in utero preleukemic clones. Home renovations and pesticide contact during pregnancy have been linked to an increased ALL risk, with subgroup analyses supporting these associations [38]. Elevated levels of organophosphate metabolites have also been reported in children with ALL compared with controls [39]. However, no interactions were observed with indoor ventilation, nor were they associated with maternal passive smoking, analgesic use, or viral infections during pregnancy [38], suggesting that the impact of certain exposures may depend on the surrounding biological and environmental context.

Similarly, studies from Brazil have associated early-onset leukemia with maternal occupational exposure to chemicals, particularly in agricultural, chemical, and petrochemical settings [40]. Although evidence linking environmental benzene exposure to pediatric leukemia remains limited, recent findings point to a potential role for this compound in regions with persistent natural or industrial pollution sources [41]. These results underscore the need for more robust epidemiological studies in pediatric populations and for public policies aimed at reducing environmental exposures as part of comprehensive prevention strategies.

With 60% of Mexico’s population enrolled at the IMSS, the institution has a unique opportunity to analyze the national epidemiological landscape and maintain a comprehensive registry for AL classification (Figure 3). Approximately 45% of pediatric oncology patients in Mexico receive treatment at the IMSS, while the remaining 55% are treated in other institutions from the federal or the private healthcare system. Through OncoCREAN, equitable access to care has been achieved, leading to a significant reduction in mortality rates, regardless of patients’ geographic location.

Since March 2022, when the OCL began operations with the reception of its first diagnostic sample, the first-year overall survival rates among patients diagnosed that year varied notably based on the type of monitoring received. Early mortality rates were lower in patients who underwent MRD testing at the OCL compared to those who did not (10.3% vs. 24.6%, *p* < 0.01). The results showed that MRD monitoring at the OCL remained a significant protective factor against early death, even after adjusting for other clinical variables (adjusted hazard ratio [aHR] 0.41; 95% CI: 0.22–0.77; and *p* < 0.01) [21].

Within the PRONAII under the OCL management, six different research groups were provided with patient samples and clinical data for their research from our PRONAII cell repository (Figure 2). To request samples and/or data, the responsible researcher must be contacted, and a confidential commitment letter must be signed [9]. The purposes of using the samples in the different projects include the evaluating new diagnostic and prognostic tools, identifying risk profiles, searching for possible therapeutic targets, conducting genomic studies, analyzing snoRNA expression, evaluating new pharmacological targets, and studying the tumor microenvironment, from the creation of bad prognosis prediction profiles to understanding how environmental contaminants affect the hematopoietic microenvironment [15,16,21,42].

The OCL is in a rural and decentralized area of the capital of Mexico, which enables an alignment with the country’s healthcare needs, where the recommendation is that samples be transported rather than patients. During the implementation of the laboratory, the main challenges have been the logistics and courier services required to ensure the timely delivery of samples to preserve their viability. Across this study, the average transport time for non-evaluable samples was 2.3 days (2022–2025), whereas a representative set of assessable samples showed an estimated transport time of 1.3 days to the OCL. These additional 24 h are critical to preserve cellular viability and enable accurate interpretation. The OCL’s efforts and efficient communication with OncoCREAN centers remain focused on reducing the proportion of non-assessable samples. The rate of non-evaluable samples for result interpretation has been decreasing over time. In 2022, samples were received from 18 states, with a non-evaluable sample rate of 1.8%. Although the sample volume increased by 211% in 2025 during the study period, the proportion of non-evaluable samples decreased to 0.7%.

Historically, pediatric cancer care in Mexico was concentrated in four major centers: two in Mexico City, one in Monterrey, and one in Guadalajara. Today, while these centers continue to operate, the system has expanded to include 32 specialized pediatric units (Figure 3). These units now provide care to patients with an established oncological diagnosis, including initial surgical interventions and induction therapy. The gradual implementation of these centers over a 24-month period has enhanced not only early diagnosis but also the close monitoring of patients, particularly in identifying therapy-resistant cells to inform clinical decisions. With the OncoCREAN strategy, 90% of diagnoses were made in less than seven days, and more than 90% of treatments started within three days of a diagnosis, which has been key to improving clinical outcomes [43]. Likewise, MRD assessments are communicated to attending physicians within 72 h, enabling timely and actionable clinical interventions: patients with detectable MRD received post-induction therapy intensification and closer monitoring, whereas patients with non-detectable MRD continued standard treatment protocols [21]. In various hematological malignancies, immunophenotyping via flow cytometry is essential for informed medical decision-making. The effectiveness of interpreting the results is inversely proportional to the variability in their presentation. To achieve uniformity in the reporting of immunophenotypes nationwide, one strategy of the OCL was to harmonize result reports by using a single reporting form [44]. This data harmonization has allowed for consistent communication among the 28 states that send samples to the laboratory, integrating the immunophenotypic information of tumor populations and thus supporting informed clinical decisions.

One of the most significant achievements has been the reduction in the abandonment of treatment rate to less than 2%, in contrast to other institutions where this figure reaches up to 40%. Furthermore, the survival rate has risen to over 84%, thanks to timely care, the effective management of complications, and the use of cutting-edge therapies [43].

Delays in diagnosis and treatment are known to significantly reduce survival rates in patients with AL. To address this, the IMSS has launched the OncoCREAN nodes, which ensure nationwide coverage for children’s health insurance. These nodes aim to guarantee timely care for suspected AL cases, reducing diagnostic delays and improving patient outcomes.

The OncoCREAN nodes are designed to improve access to care in remote regions, optimize resource coordination, and standardize procedures across the country. Furthermore, the data collected from these nodes will enable the creation of regional maps to achieve the following

(a) Identify geographic patterns to visualize areas with high incidence rates, potentially uncovering environmental or socioeconomic factors contributing to the disease.
(b) Facilitate epidemiological research to gain a deeper understanding of the causes and risk factors associated with childhood leukemia, leading to more effective prevention strategies.
(c) Raise public awareness about childhood leukemia and emphasize the importance of continuing education at multiple levels for early detection and appropriate treatment.
(d) Evaluate the effectiveness of public health interventions and programs in different regions, improving control and treatment strategies.

It is well known that health strategies can have varied impacts within the same country due to regional socioeconomic complexity, the health infrastructure, and public policies. Our group has identified epidemiological differences across different regions and states, as highlighted by López-Aguilar et al. [13], showing a higher incidence of B-cell ALL (Figure 4).

Consequently, local strategies, such as the twinning program between Rady Children’s Hospital in San Diego (United States) and Tijuana General Hospital (Mexico), have been implemented in Baja California by other groups. This program aims to assess its impact on building sustainable, high-quality care capacities and improving leukemia survival rates in children from LMICs. The result has been a 5-year overall survival improvement from 59% to 65% [45]. These collaborative models highlight the importance of support networks, including service delivery and workforces, in effectively and sustainably improving survival outcomes for children with ALL at the US–Mexico border. However, data from Dr. Jose Eleuterio Gonzalez University Hospital reveal that no improvement in survival rates was achieved for children with ALL from low-income families living in LMICs when treated with two consecutive 5-year protocols at a referral center in northeastern Mexico [46]. This underscores the urgent need to implement multi-level, integrative strategies that focus on timely diagnosis and appropriate early treatment for Mexican children and adolescents with AL. Improving survival and quality of life for children with cancer in Mexico and similar settings requires coordinated actions [47]. As in other LMICs, urgent investments in infrastructure, medical equipment, professional training, and diagnostic and treatment capacity are essential [4]. Evidence from Brazil demonstrates that establishing dedicated pediatric oncology units can increase survival from 32% to 63% [47,48,49].

Cell repositories play a crucial role in advancing biomedical research, as scientific accuracy and ethical rigor depend on proper sample collection, storage, and documentation [50,51]. Since we received samples from all over the country, ethical approval from patients and legal tutors to keep their samples in storage was a major challenge. Social service medical interns are required to collect informed consent within the OncoCREAN units. So a sample stored in our repository must fulfill these requirements. Implementing structured sample storage policies and infrastructure is crucial for maintaining scientific quality, ensuring regulatory compliance, and promoting responsible research conduct [51].

This led to the designation of the OCL as the reference laboratory for all OncoCREAN centers, supported by standardized diagnostic protocols, periodic epidemiological reporting, and multidisciplinary case discussions to optimize patient management.

Recent advances in image processing and artificial intelligence (AI) algorithms have improved cancer diagnosis and prognosis, enabling highly precise tumor detection and the faster interpretation of imaging and tissue samples [52]. Some examples include melanoma, where skin lesion classification has a major role in the early and accurate diagnosis of skin cancer, and new specialized models based on dermoscopic images have been developed [53]; another important model is the Multimodal transformer with Unified maSKed modeling (MUSK), which predicts relapses, pan-cancer prognosis, and immunotherapy responses [54].

In leukemia, predictive efforts have focused on liquid samples; the DDPR model, for instance, estimates the relapse risk at B-ALL diagnosis using mass cytometry [55]. In a collaborative effort with the K Davis group from Stanford University, we are currently working on a predictive model adapted to our country, including other parameters like the tumor microenvironment. Given regional differences in genetics, environments, and lifestyles [52], integrating our data into global knowledge networks is essential to evaluate new technologies and inform countries with socioeconomic and exposure conditions like Mexico.

To date, there are no sufficiently defined environmental targets that would allow for the design of specific regional policies with a clearly demonstrated impact on the incidence of childhood acute leukemia, since isolated associations do not necessarily imply causality. Given that ionizing radiation remains the only environmental agent with solid causal evidence, public policy should focus on strategies aimed at minimizing unnecessary exposures, particularly occupational exposure, as well as strengthening early detection and reducing regional inequalities in access to diagnosis and treatment. In line with Greaves’ proposal, the progress in understanding the causes of ALL should be supported by a deep understanding of cancer biology and the integration of multidisciplinary research and international consortia, with the goal of informing more robust public health policies and the development of effective preventive interventions.

In this context, the accumulated knowledge on contributing environmental factors in the etiology of pediatric leukemia, integrated with the OncoCREAN epidemiological registry, represents a strategic opportunity to contribute to the design and strengthening of public policies in Mexico. The availability of high-quality clinical, sociodemographic, and immunophenotypic data will allow not only the description of the disease burden but also the analysis of spatial and temporal patterns of incidence, as well as its possible association with relevant environmental exposures in different geographic areas of the country.

The OncoCREAN registry offers a unique platform to move from isolated associations to integrative models that consider the interaction between environmental, biological, social, and developmental factors. In a country with significant geographic, socioeconomic, and environmental heterogeneity like Mexico, this approach will be innovative for identifying vulnerable pediatric populations and risk scenarios, generating essential local evidence for public health decision-making.

Finally, the use of the OncoCREAN registry as a tool for translational research and epidemiological surveillance can help bridge the gap between the generation of scientific knowledge and its application in public policy. In this way, the IMSS can play a key role not only in the care and treatment of pediatric leukemia patients but also in developing national strategies for prevention, ensuring equitable access to care and reducing the burden of childhood cancer in Mexico.

## 5. Conclusions

The OncoCREAN model demonstrates that decentralizing high-technology diagnostic resources is a critical requirement for modern pediatric oncology. Anchored by specialized centers such as the OCL, this institutional framework proves that rapid, high-precision diagnoses can be achieved beyond major metropolitan hubs. More than improving immediate survival rates, this infrastructure generates a powerful engine for translational medicine, providing essential data on disease incidence and molecular profiles that inform national health policies. This implementation represents a transformative shift in pediatric oncology, moving beyond traditional centralized care toward a robust interinstitutional network. By integrating high-technology nodes, this approach bridges the gap between advanced science and patient access, optimizing diagnostic timelines while serving as a vital platform for precision medicine, real-time incidence monitoring, and personalized therapeutic strategies. Crucially, the model also emphasizes collaboration with local healthcare providers through the establishment of networks with hospitals and professionals to ensure effective referral pathways and follow-up care based on field diagnoses. In parallel, community and patient education initiatives are essential to promote the benefits of early diagnosis and the use of high-tech tools, while addressing cultural and language barriers that may hinder understanding and acceptance. Ultimately, by deploying sophisticated resources closer to vulnerable populations, the model fosters health equity, strengthens clinical outcomes, and establishes a scalable blueprint for reducing diagnostic delays in low- and middle-income regions. Ensuring sustainability and scalability will be paramount to guarantee that this initiative remains viable long-term and can be expanded to reach more regions and patients across Mexico, consolidating a new standard of care for pediatric cancer patients in both rural and urban areas.

## Data Availability

The datasets generated and/or analyzed during the current study are not publicly available due to patient confidentiality and ethical restrictions. Although anonymized identifiers (acronyms) were used, the underlying clinical data cannot be shared to protect participant privacy.

## Supplementary Materials

The following supporting information can be downloaded at: https://zenodo.org/records/18393973 accessed on 19 December 2025. Supplementary Information: Guide for Sample Shipment: Immunophenotype and Minimal/Measurable Residual Disease Study.

## Abbreviations

The following abbreviations are used in this manuscript:

aHR	Adjusted Hazard Ratio
AI	Artificial Intelligence
AL	Acute Leukemia
ALAL/MPAL	Acute Leukemia of Ambiguous Lineage/Mixed Phenotype Acute Leukemia
ALL	Acute Lymphoblastic Leukemia
AML	Acute Myeloid Leukemia
B-ALL	B-Cell Precursor Acute Lymphoblastic Leukemia
BM	Bone Marrow
CI	Confidence Interval
COG	Children’s Oncology Group
CONAHCYT	National Council of Humanities, Sciences, and Technologies
DDPR	Developmentally Dependent Predictor of Relapse
GICC	Global Initiative for Childhood Cancer in Latin America
GNI	Gross National Income
HICs	High-Income Countries
HR	Hazard Ratio
ICU	Intensive Care Unit
IMSS	Mexican Social Security Institute
LMICs	Low- and Middle-Income Countries
MDSs	Myelodysplastic Syndromes
MRD	Measurable Residual Disease
MIC	Middle-Income Country
MUSK	Unified maSKed Modeling
NGOs	Non-Governmental Organizations
OCL	Oncoimmunology and Cytomics Laboratory
OncoCREAN	Statal Reference Centers of Pediatric Oncology
OR	Odds Ratio
PINDA	Programa Infantil Nacional de Drogas Antineoplásicas
PRONAII	National Project for Research and Incidence
SES	Socioeconomic Status
SOPs	Standardized Operating Procedures
T-ALL	T-Cell Acute Lymphoblastic Leukemia
